# Medicinal Shock—Arnica

**Published:** 1891-06

**Authors:** Wm. Jefferson Guernsey

**Affiliations:** Frankford, Philada.


### MEDICINAL SHOCK—ARNICA.

WM. JEFFERSON GUERNSEY, M. D., FRANKFORD, PHILADA.

It was my misfortune, in the winter of 1882, to suffer from an agonizing odontalgia. The pain was so intense as to drive me from the office in desperation. As I seemed utterly unable to think what medicine I needed, one of my family suggested the application of Arnica tincture, and it was rubbed upon the gum and teeth for about fifteen minutes without effect. After suffering for an hour my wife called attention to my peculiar position, and it was the only one I could tolerate, namely, bending *my head down as low as possible.* I was doubled over so as to have my head nearly touch the floor. Although stupefied with pain, I managed to refer the symptom to Arnica, "lies preferably with the head low;" not an exact description, to be sure, but a similarity in the condition of amelioration. Besides this I had an inordinate dread of having any one approach me, and was particularly afraid that my child would "run into me." Here was a second strong indication of the remedy and Hering gives "excruciating pain * * * right, upper jaw," the exact location. I dissolved a few globules of Arn.$^{45m}$ (Fincke) in water and on taking a teaspoonful, felt, *at once* a marked "*shock*" as from electricity through the entire body, and the pain was entirely gone. The transition to freedom from suffering was indescribable. I was afraid to move. After assuring myself that the relief was real, and enjoying about twenty minutes of bliss, I felt a slight, dull ache in the teeth, and in five minutes more the principal tooth had gotten pretty well down to business again.

Another teaspoonful produced the same "shock" and was followed by the same instantaneous relief, but after a second respite of about the same duration, the programme was repeated. This third dose would doubtless have removed the trouble permanently, had not a little foolish curiosity got the better of me.

After tea, while resting in a reclining chair, I was amused to find that I could *produce* a slight, dull pain in the tooth by assuming the prone position; strange as it seemed, having my head at all low now caused the pain. While thus experimenting too long, I found my tooth really aching some. Instead of sitting up and waiting patiently, I took another teaspoonful of the solution. Now mark the result: the pain returned in all its old severity and with it the absolute necessity to take the clownish position of standing on my head, or nearly so. Feeling assured that the extra dose had caused the relapse, I determined to wait an hour or two before taking anything else. This was not necessary, for in half an hour the pain *gradually* abated and I fell asleep to wake an hour later without it. The next day a dentist satisfied me that I had not had an ordinary toothache from cold alone by plunging into an exposed nerve.

It is worthy of note that the pain was benefited by the 45M *Fluxion* potency while the tincture had failed to aid in the least when applied, or when swallowed (for some of it did pass into the stomach with saliva).

Turning to the drug in Allen's *Encyclopedia*, we find, p. 491, "Jerks and shocks in the body, as by electricity," showing that the sensation which preceded the rapid amelioration was undoubtedly a proving. I recollect having heard the expression from others, who had experienced the symptom on taking medicine, but do not know the drugs under which it occurred.

